# Treatment of Malignant Tumors by the Injection of Oil of Turpentine

**Published:** 1886-08

**Authors:** 


### *TREATMENT OF MALIGNANT TUMORS BY THE INJECTION OF OIL OF TURPENTINE.*

Turpentine injected into the tissues excites a violent inflammation. Alcohol, under the same circumstances, will produce a contraction of the tissues, thus favoring absorption. With these facts as a basis, Vogt determined to treat malignant tumors which could not be conveniently operated on, by injecting into them a mixture, composed of two parts of alcohol to one of turpentine. In this manner, he has treated two cases of cancer of the breast, one case of lymphosarcoma of the axilla and one of multiple sarcoma. One-half to one-third of a Pravaz syringeful is injected. This produces inflammation with fever and other constitutional symptoms. In from six to eight days, the tumor either becomes harder and is reduced in size, or is softened and eliminated.—*Il Raccoglitore Med.*

